# Age estimation from mandibles in Malay: A 2D geometric morphometric analysis

**DOI:** 10.1016/j.jtumed.2023.05.020

**Published:** 2023-06-07

**Authors:** Nur Ariessa Farhana Zulkifli, Nur Aliya Syuhada Mohd Saaid, Aspalilah Alias, Nurjehan Mohamed Ibrahim, Choy Ker Woon, Arofi Kurniawan, Beshlina Fitri Widayanti Roosyanto Prakoeswa

**Affiliations:** a Department of Basic Sciences and Oral Biology, Faculty of Dentistry, Universiti Sains Islam Malaysia, Malaysia; b Department of Forensic Odontology, Faculty of Dental Medicine, Universitas Airlangga, Indonesia; c Forensic Odontology Unit, Department of Imaging & Pathology, KU Leuven, Belgium; d Department of Orthodontic and Paediatric Dentistry, Faculty of Dentistry, Universiti Sains Islam Malaysia, Malaysia; e Department of Anatomy, Faculty of Medicine, Universiti Teknologi MARA, Sungai Buloh Campus, Selangor, Malaysia

**Keywords:** Age estimation, Dental panoramic tomography, Geometric morphometric, Identification, Mandible, الفك السفلي, تقدير العمر, التصوير المقطعي المقلاعي السني, المورفومتريا الهندسية, التعريف

## Abstract

**Objectives:**

In this study, the sizes and forms of mandibles in various age groups of the Malay population were measured and compared.

**Methods:**

Geometric morphometric (GM) analysis of mandibles from 400 dental panoramic tomography (DPT) specimens was conducted. The MorphoJ program was used to perform generalized Procrustes analysis (GPA), Procrustes ANOVA, principal component analysis (PCA), discriminant function analysis (DFA), and canonical variate analysis (CVA). In the tpsDig2 program, the 27 landmarks were applied to the DPT radiographs. Variations in mandibular size and form were categorized into four age groups: group 1 (15–24 years), group 2 (25–34 years), group 3 (35–44 years), and group 4 (45–54 years).

**Results:**

The diversity in mandibular shape among the first eight principal components was 81%. Procrustes ANOVA revealed significant shape differences (P < 0.001) among age groups. Mahalanobis distances indicated substantial differences among all age groups; group 1 and group 4 scored highest, at 2.114. The ranges for the cross-validation and discriminant function tests were 90–72% and 81–49%, respectively.

**Conclusion:**

GM analysis through radiography is a simple, non-invasive, and non-destructive method of estimating age by using the mandible. GM analysis is unique because it can visualize the changes in mandible shape among age groups. This method should aid in age identification in forensic odontology investigations.

## المخلص

**أهداف البحث:**

تهدف هذه الدراسة إلى قياس ومقارنة حجم وشكل الفك السفلي في مجموعات عمرية مختلفة من السكان الماليزيين.

**طرق البحث:**

أجري تحليل المورفومتريا الهندسية للفك السفلي باستخدام 400 عينة من التصوير المقطعي المقلاعي السني. استخدم برنامج مورفوجي لإجراء تحليل بروكرست العام، وتحليل تباين بروكرست، وتحليل المكونات الرئيسية، وتحليل الدالة التمييزية، وتحليل المتغيرات الكنسية. تم تطبيق ما مجموعه 27 علامة بارزة على صور التصوير المقطعي المقلاعي السني باستخدام برنامج "ت ب اس د آي ج 2". لتصنيف التغيرات في حجم وشكل الفك السفلي، تم إنشاء أربع مجموعات عمرية: المجموعة 1 (15-24 عاما)، والمجموعة 2 (25-34 عاما)، والمجموعة 3 (35-44 عاما)، والمجموعة 4 (45-54 عاما).

**النتائج:**

أظهرت التغييرات في أشكال الفك السفلي متغيرا بنسبة 81% على أساس المكونات الرئيسية الثمانية الأولى. كشف تحليل تباين بروكرست عن اختلافات كبيرة في الشكل بين المجموعات العمرية. أظهرت مسافات ماهالانوبيس اختلافات كبيرة بين جميع المجموعات العمرية، حيث سجلت المجموعة 1 والمجموعة 4 أعلى قيمة بـ 2.114. تراوحت نسب الصحة لاختبارات التحقق المتقاطع بين 90% و72% ولاختبارات الدالة التمييزية بين 81% و49%.

**الاستنتاجات:**

في الختام، يعتبر استخدام الأشعة السينية لتحليل المورفومتريا الهندسية طريقة بسيطة وغير ضارة وغير تدميرية لتقدير العمر باستخدام الفك السفلي. يعتبر تحليل المورفومتريا الهندسية فريدا لأنه يمكن تصوير تغييرات شكل الفك السفلي بين المجموعات العمرية المختلفة. تعتبر هذه الطريقة مفيدة لتحديد العمر في التحقيقات الطبية الشرعية في طب الأسنان.

## Introduction

The basic approach to identifying individuals involves estimating individual characteristics such as age, sex, stature, and ancestry.^1^ Estimating the age of living individuals is essential in tracking immigrants' movements when valid identity documents are unavailable and assessing their status in criminal cases.^2^ Generally, forensic dentists attempt to determine the general physical descriptions of remains, including age, sex, race, cause, manner of death, any individualistic features, and an estimate of the time since death.^3^

As adults mature, all their bones stop growing, thus making age estimation difficult. Forensic studies on age estimation in living individuals have been a research focus for many years.^4^ Forensic study also encompasses the realm of competitive sports and legal and refugee issues.^5^ The most precise method for determining age is dental examination. Age is strongly associated with dental maturation. The teeth are among the most robust and explosive-resistant bones in the body. The chronological development of dental growth is crucial for determining age.^6^

Forensic dentists use the maxilla, mandible, and teeth to assess odontological characteristics, because they remain intact in intensely damaged bodies.^3^ The mandible is the bone most often found in human remains and is sometimes the only bone available to perform post-mortem investigation, particularly after fires and explosions.^7^ The mandible is an excellent bone for estimation of a person's age. In both morphological and dimensional parameters, it shows more pronounced development than the other facial bones and teeth. The dimensions of the mandible—particularly the mandibular angle, mandibular ramus height, and bigonial breadth—have been suggested to strongly correlate with human age.^8^ The masseter, the medial and lateral pterygoid, the temporalis, and the masticator are the primary muscles used in chewing. Knowing the anatomy of these muscles, their attachments, and how they work is essential, because they can alter the mandible's appearance. In some populations, individuals of different ancestries, sexes, and ages have different customs, lifestyles, and eating habits.^9^

The radiological procedure has several advantages over histology and biochemical methods, among the various procedures used to determine a person's sex, ancestry, and age.^10^ For identifying remains, the radiography approach is easy, rapid, affordable, and non-invasive.^11^ To identify each person, a radiographic scan is used to ascertain the age, sex, race, stature, and cause of death.^12^ Conventional radiographs are used for the comparative identification of antemortem and post-mortem interventions by allowing for observation of anatomical characteristics, such as coronal shape and size, pulp structure, location, and the shape of the alveolar bone crest.^13^ Dental panoramic tomography (DPT) is are among the dental radiological methods used by dentists. DPT can identify many structures of the mouth and teeth, and enables objective and reproducible collection of 2D images to analyze human variation.^14^

To differentiate the biological profiles of skeletal remains, forensic anthropologists have used both qualitative and quantitative methods.^15^ The two types of morphometric analysis used in forensic anthropology are traditional and geometric.^5^ Modern digital software is used to digitally map measurements obtained with calipers during traditional morphometric examination, to calculate linear metric distances. To evaluate biological identity in this anatomical location, a novel technique known as geometric morphometric (GM) analysis was developed.^5^ In this approach, statistical methods can be used to investigate the patterns of individual variation. The measurements may reveal bone size and form. If the traditional method is used on big data, the analysis becomes complicated.^16^ Traditional morphometrics provides only scatter plots or numerical representations of statistical connections, but not assessments of the forms themselves.^17^ Thus, researchers have modified their strategies to adopt more complex GM studies.^5^ GM involves the statistical analysis of form according to Cartesian landmark coordinates.^17^ The landmarks can be recorded as two- or three-dimensional coordinates, thus resulting in a spatial framework of the chosen points and allowing for statistical analysis through various transformative or computational methods.^5^

GM comprises a collection of analytical techniques and procedures.^18^ The use of digital techniques is becoming increasingly common in dental radiology, owing to advances in diagnostic performance, decreased patient doses, and financial benefits.^19^ Through the use of GM analysis, this project created a Malay population database derived from various DPT landmarks to aid in future forensic identification of Malay individuals. After this study, this database may be used as input to create a cross-platform software for forensic odontology applications of age estimation in human identification.

## Materials and Methods

The study was carried out at the Dental Clinic at Universiti Sains Islam Malaysia (USIM). The USIM Ethics Committee granted ethical approval (number USIM/JKEP/2020-90). Four hundred images from the USIM dental clinic's 2013–2020 database of DPT radiographs obtained through Planmeca Romexis software composed the study sample. Radiographs of patients meeting the inclusion criteria were selected and analyzed. The patients included male and female individuals of Malay ethnicity in different age groups. Patients with loss of more than eight teeth at the mandible, presence of bone resorption at the mandibles which more than 1.0 (according to periodontal risk assessment), any history of mandibular surgery, and any other severe developmental disturbances were excluded from the study. Age, sex, and race were documented for each specimen.

**Figure 1:**
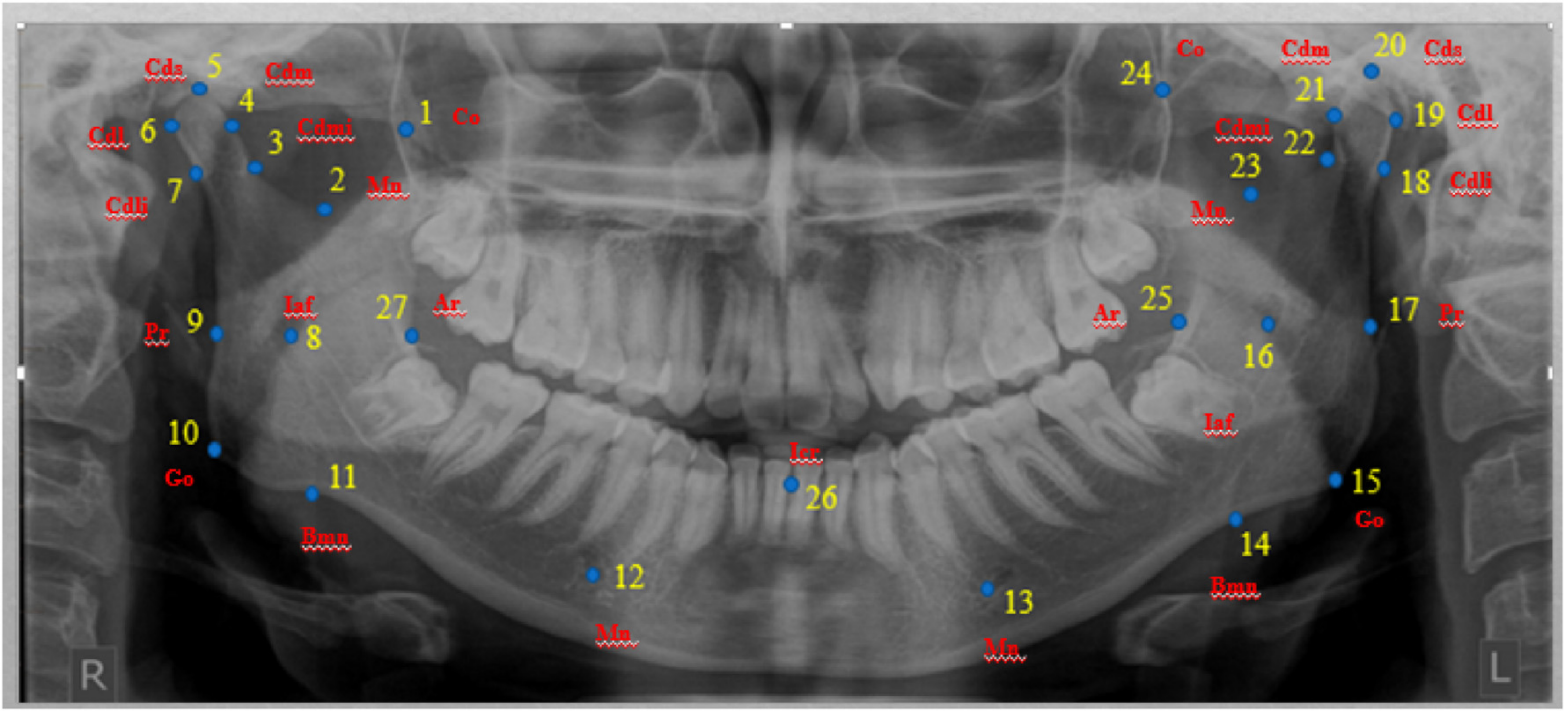
Landmarks of mandibles on DPT. For explanation of the numbers, see Table 1.

The following age groups were created according to the results: group 1 (15–24 years), group 2 (25–34 years), group 3 (35–44 years), and group 4 (45–54 years). A total of 400 DPT radiographs from 100 Malay patients from each of the four groups were analyzed. For all analyses, data for 286 females and 95 males were pooled. The data for 400 patients undergoing DPT at the Faculty of Dentistry, USIM, were extracted with Planmeca Romexis software; tpsDig2 (version 2.31) was used to apply landmarks during the GM analysis, and MorphoJ (version 1.07a) was used to analyze the data. The SPSS version 23 program was used to analyze quantitative data. A total of 27 2D hard tissue landmarks were applied to the mandibles in this investigation (Table 1, Figure 1).

**Table 1:** Definitions and numbers of landmarks of mandibles in DPT.

No.	Landmarks	Definition
1	Coronion (Co)	The most superior point on the coronoid process (right)
2	Mandibular notch (Mn)	The most inferior point on the mandibular notch (right)
3	Condylion medial inferialis (Cdmi)	Medial point on the mandibular condyle located at the most curved area, horizontally aligned with landmark no. 7 (right)
4	Condylion mediale (Cdm)	The most medial point on the mandibular condyle (right)
5	Condylion superior (Cs)	The most superior point on the mandibular condyle (right)
6	Condylion laterale (Cdl)	The most lateral point on the mandibular condyle (right)
7	Condylion lateral inferialis (Cdli)	Lateral point on the mandibular condyle located at the most curved area, horizontally straight with landmark no. 3 (right)
8	Inferior alveolar foramen (Iaf)	The most inferior point on the margin of the inferior alveolar foramen (right)
9	Posterior ramus (Pr)	Single point located at the posterior border of the ramus, horizontally straight with landmarks no. 8 and 27 (right)
10	Gonion (Go)	The most lateral external junction point of the horizontal and ascending rami of the lower jaw (right)
11	Body mandibular notch (BMN)	The deepest groove in the ramus of the mandible (right)
12	Mentale (Ml)	The most inferior point on the margin of the mandibular mental foramen (right)
13	Mentale (Ml)	The most inferior point on the margin of the mandibular mental foramen (left)
14	Body mandibular notch (BMN)	The deepest part of the mandible body (left)
15	Gonion (Go)	The most lateral external junction point of the horizontal and ascending rami of the lower jaw (left)
16	Inferior alveolar foramen (Iaf)	The most inferior point on the margin of the inferior alveolar foramen (left)
17	Posterior ramus (Pr)	Single point located at the posterior border of the ramus, horizontally straight with landmarks no. 16 and 25 (left)
18	Condylion lateral inferialis (Cdli)	Lateral point on the mandibular condyle located at the most curved area, horizontally straight with landmark no. 22 (left)
19	Condylion laterale (Cdl)	The most lateral point on the mandibular condyle (left)
20	Condylion superior (Cs)	The most superior point on the mandibular condyle (left)
21	Condylion mediale (Cdm)	The most medial point on the mandibular condyle (left)
22	Condylion medial inferialis (Cdmi)	Medial point on mandibular condyle located at the most curved area, horizontally straight with landmark no. 18 (left)
23	Mandibular notch (Mn)	The most inferior point on the mandibular notch (left)
24	Coronion (Co)	The most superior point on the coronoid process (left)
25	Anterior ramus (Ar)	Point at which the minimum breadth transects the anterior border of the ramus, horizontally straight with landmarks no. 16 and 17 (left)
26	Incisor (Icr)	Midpoint located between two mandibular central incisors
27	Anterior ramus (Ar)	Point at which the minimum breadth transects the anterior border of the ramus, horizontally straight with landmarks no. 8 and 9 (right)

The landmarks' 2D coordinates were analyzed in the shape analysis program MorphoJ. For removal of non-shape variation from the sample, the raw landmark coordinates from each mandible in the DPT were first examined with generalized Procrustes analysis (GPA). This procedure involved scaling, rotating, and translating methods. To use each mandible in the DPT as a biologically valid measurement of the overall size of the landmark configuration of the hard tissue, the scaling technique modified the landmark coordinates to ensure that each mandible had a unit centroid size. The data were grouped and arranged according to shape similarity through principal component analysis (PCA). Wireframe and principal component (PC) plots were used to analyze and visualize the form differences revealed by PCA. For grouped data, canonical variate analysis (CVA) was used. On the basis of sample centroids, CVA calculates Mahalanobis distances between groups. Discriminant function analysis (DFA) and cross-validation were used to evaluate classification accuracy. The PC scores from the samples' GPA/PCA were used in both analyses. For quantitative analysis, data from MorphoJ were exported to SPSS software.

## Results

**Figure 2:**
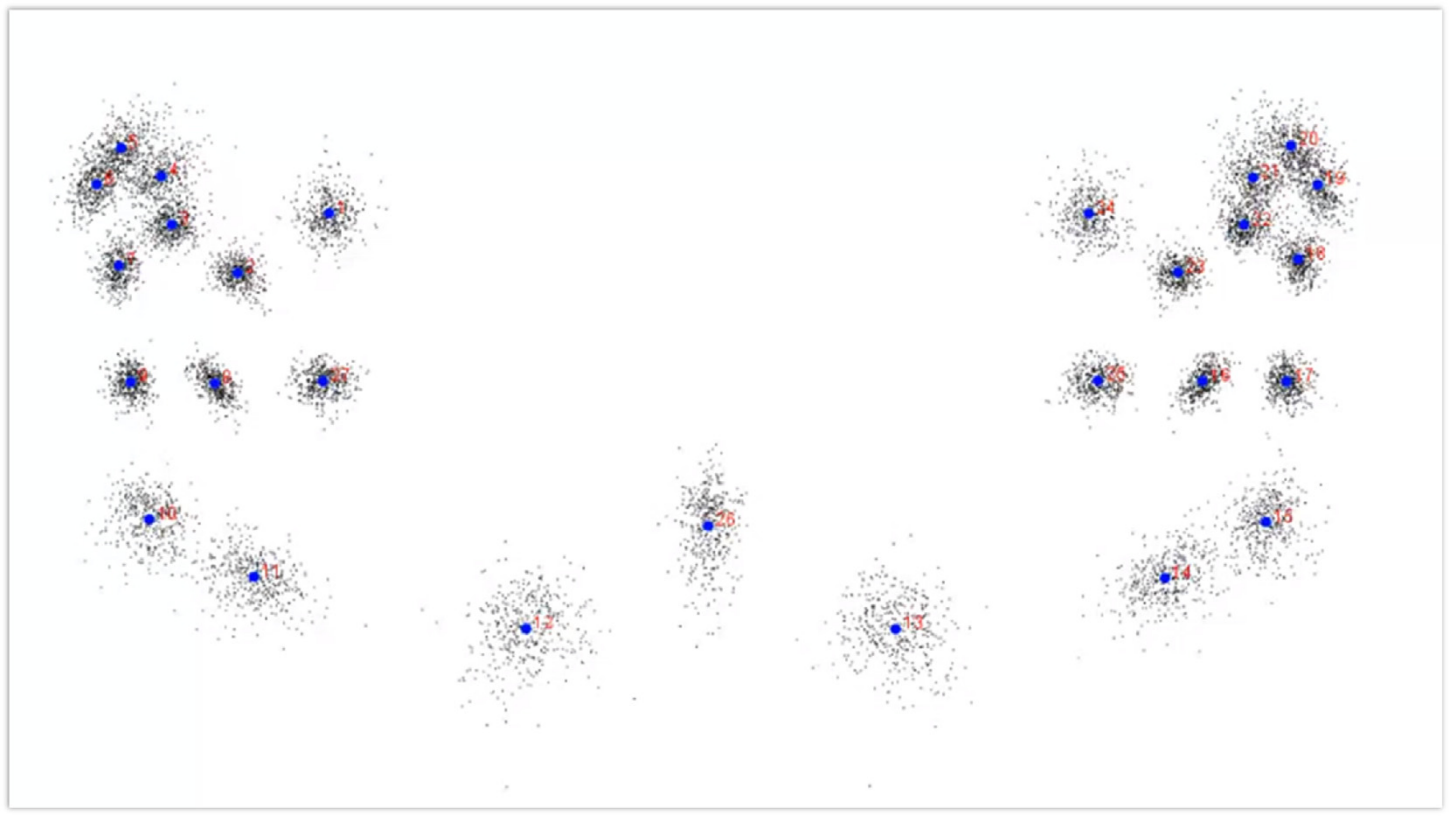
Generalized Procrustes analysis (GPA) consisting of a scatterplot of the superimposed landmark configurations, with 27 landmarks placed in 400 DPT images.

A new matrix of Procrustes coordinates was generated by the GPA, which superimposed each group of landmarks before scaling and rotating to a centroid size. The scatterplot in Figure 2 displays the placed configurations of 27 landmarks from GPA, indicating the morphological forms of all 400 DPT images. The black dots show the landmark positions for specific configurations within the samples, and the blue dots indicate the mean landmark positions.

**Figure 3:**
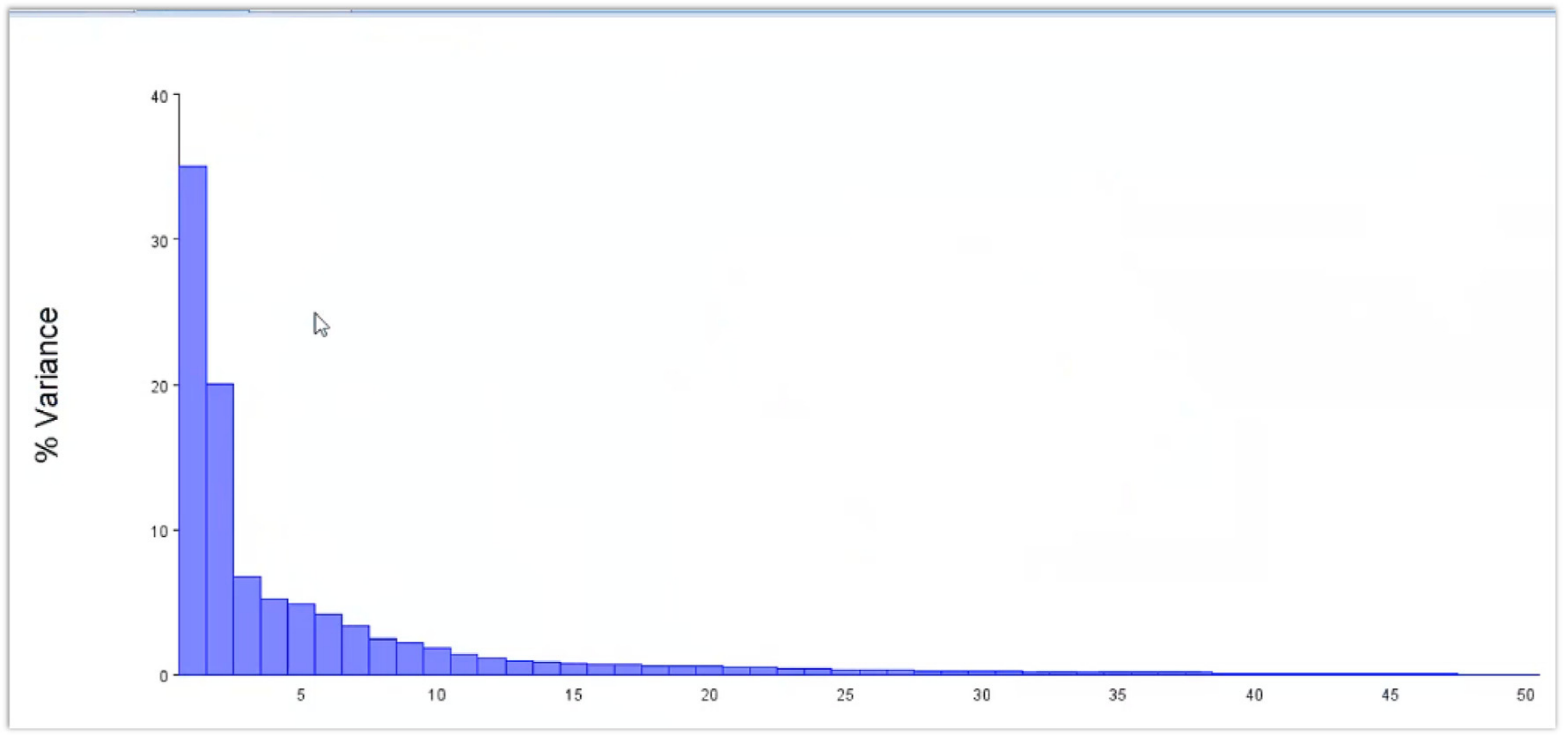
Screen plot showing the amount of variance for all samples.

Multivariate analysis and the main components of shape variation in the data set were revealed by PCA. The variation for each age group is depicted in Figure 3. The first eight principal components, with PC1 = 35%, PC2 = 20%, PC3 = 6.7%, PC4 = 5.2%, PC6 = 4.2%, PC7 = 3.3%, and PC8 = 2%, showed 81% variance in mandible form. Table 2, Table 3, Table 4, Table 5 demonstrate, by age group, how the wireframe of PC1 through PC3 varied in shape. Dark blue represents the distinct changes in different PCs and age groups, whereas light blue indicates the average form.

**Table 2:** Wireframes from the first three principal components of group 1 (15–24 years old), exhibiting 64% variation in mandible shape.

PCA	Wireframe (group 1, 15–24 years old)
PC1 (33%)	
PC2 (22%)	
PC3 (9%.)

**Table 3:** Wireframes from the first three principal components of group 2 (25–34 years old), exhibiting 60.7% variation in mandible shape.

PCA	Wireframe (group 2, 25–34 years old)
PC1 (31.4%)	
PC2 (21.3%)	
PC3 (8%)

**Table 4:** Wireframes from the first three principal components of group 3 (35–44 years old), exhibiting 67% variation in mandible shape.

PCA	Wireframe (group 3, 35–44 years old)
PC1 (41%)	
PC2 (19%)	
PC3 (7%)

**Table 5:** Wireframes from the first three principal components of group 4 (45–54 years old), exhibiting 62% variation in mandible shape.

PCA	Wireframe (group 4, 45–54 years old)
PC1 (38%)	
PC2 (17%)	
PC3 (7%)	

Separate ANOVA tables for centroid size and shape display the results of the Procrustes ANOVA (Table 6). No significant changes in centroid size were found, although significant differences in centroid shape (P < 0.0001) were observed throughout each age group.

**Table 6:** Centroid size and shape: sum of squares (SS), mean square (MS), degrees of freedom (df).

Effect	SS	MS	df	F	P
Centroid size	112262.9989	37420.9989	3	0.55	0.6456
Shape	0.0165	0.00011	150	2.13	<0.0001∗

∗P < 0.05, significant difference.

**Figure 4:**
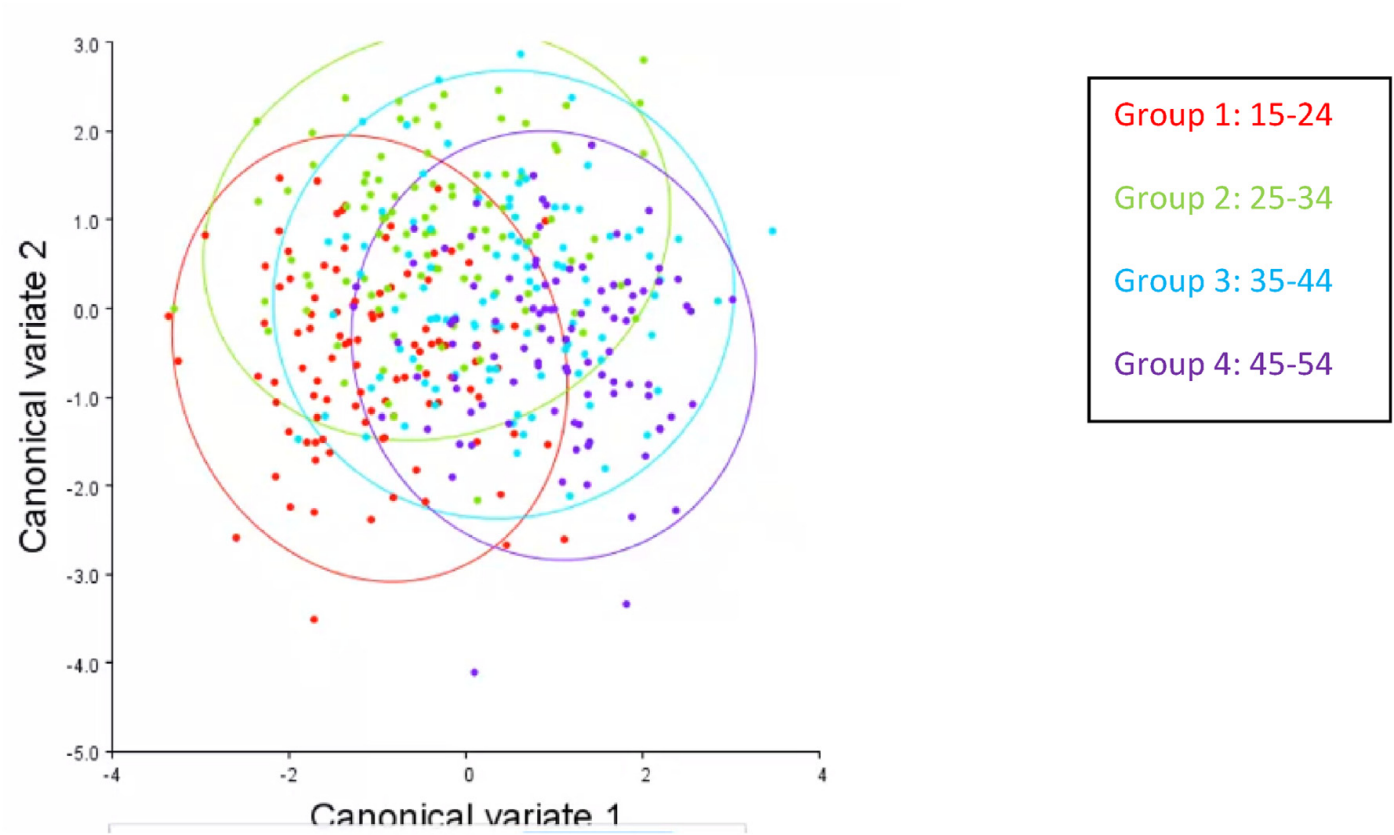
Canonical variates of shape, with four distinguishable groups showing some overlap.

Figure 4 illustrates the overlap among age categories with CVA. Group 1 (15–24), group 2 (25–34), group 3 (35–44), and group 4 (45–54) are represented by red, green, blue, and purple, respectively. Two distances were involved in CVA: Procrustes distances (size) and Mahalanobis distances (shape). Mahalanobis distances revealed substantial differences in all group ages, with group 1 vs group 4 scoring 2.114, and group 3 vs group 4 scoring 1.269. Group 1 vs group 4 had the highest score (0.0148), and group 3 vs group 4 had the lowest score (0.006). The Procrustes distances showed significant differences in only group 1 vs groups 2, 3 and 4 (P < 0.05), whereas no significant differences were observed in the comparisons among other groups (group 2 vs group 3; group 2 vs group 4; and group 3 vs group 4).

In the DFA, group 1 vs group 4 showed the highest percentages (88% and 79% after cross-validation). In contrast, group 3 and group 4 had the lowest rate (72.5% and 53% after cross-validation). Groups 2 and 3 also showed discriminant function below 80%, which decreased to approximately 61% after cross-validation. In comparison, the other groups showed percentages of more than 60% after cross-validation of the discriminant function test (Table 7).

**Table 7:** Discriminant function analysis and cross validation among groups.

Groups	Discriminant function analysis	Cross validation
Group 1 and group 2	81%	63%
Group 1 and group 3	83%	68.5%
Group 1 and group 4	88%	79%
Group 2 and group 3	76.5%	61%
Group 2 and group 4	85.5%	71.5%
Group 3 and group 4	72.5%	53%

## Discussion

In civil, criminal, forensic, and anthropology disciplines, age estimation is crucial.^20^ Age estimation in bones is performed through a variety of techniques. Accurate age assessment is critical in forensics for unidentified remains, bodies recovered from disasters, refugees, and asylum seekers without identifying documents, as well as for legal reasons. A radiographic method or a direct bone examination can be used to estimate age. Numerous methods have been used, including X-ray, CT scanning, ultrasound, and magnetic resonance imaging.^21^ Forensic age assessment typically uses morphological techniques based on radiological analysis of the teeth and skeletal development.^1^

Because most adult teeth have already erupted, estimating an adult's age may be challenging. For age estimates in every group, applying population-specific reference standards is essential for accuracy.^22^ The facial skeleton develops throughout a person's life, and the growth rates of various facial regions vary. Clinical evidence suggests that some people's mandibles may change in shape as they age. In cosmetic surgery, any alteration to the mandible's size and shape is of utmost importance.^23^

This research focused on developing a novel approach for future forensic use in determining age range according to the morphological structure of the mandibles observed in dental radiographs, specifically those obtained through DPT. A total of 400 DPT radiographs of Malay patients were divided into four groups with different age ranges, according to a previous study.^23^ Those radiographs provided anatomical information about the entire mandible. The coordinates of the landmarks were analyzed with the GM method to determine any significant differences in size and shape among groups. The results of age estimation were relatively accurate and did not rely on prior knowledge of the sex of an individual. In this study, significant differences in the size and shape of the mandibles were observed among the four age groups. The most apparent difference was between group 1 (15–24 years old) and group 4 (45–54 years old), with 88% after DFA and 79% after cross-validation. Meanwhile, the least apparent difference was observed between group 3 (35–44) and group 4 (45–54), at 72.5% and 53% after DFA and cross-validation, respectively.

A significant difference in size and shape was observed, because the mandible continues to change during the life course. Some parts of the mandible bone may grow faster than other parts. Moreover, as age increases, the mandible width and height also increase.^24^ In addition, bone density and increased bone resorption occur with age. Consequently, the mandibular bone height decreases, and changes in its shape and size can be observed. However, our results indicated that the differences in size and shape between ages 35–44 and 45–54 were not remarkable. In adults and in older people, tooth loss and resorption rates affect changes in mandible shape.^25^

During childhood, the shape and size of the mandible also change. The length of the mandibular body increases, particularly in the area posterior to the mental foramen, to provide space for teeth to erupt. The depth also increases, because bone growth occurs at the alveolar ridge level. As an individual approaches adulthood, the size of the ramus and body of the mandible become more prominent than in the childhood phase. The condylar process becomes higher than the level of the coronoid process. The mental foramen is situated in the middle of the mandible's upper and lower borders. As age increases, the mandible dramatically decreases in size, owing to tooth loss and alveolar ridge resorption. The condylar process may be bent backward at extreme ages. Changes also occur because of increased resorption of the alveolar ridge; the mental foramen is near the upper border of the mandible. The mandibular angle is greater in older than younger people.^26^

Previous studies have used various prominent landmarks on the mandible that have great potential in age estimation in forensic analysis. The changes in morphological features on the mandible aid in estimating the age of an individual.^27^ The gonial angle and antegonial region on the mandible are among the most crucial landmarks associated with age.^28^ In adulthood, the gonial angle decreases and becomes less obtuse with increasing age. These changes have been reported to occur until 55 years of age in both men and women.^28^ Another study has found that ramus height and mandibular body length strongly correlate with an individual's chronological age; however, it has also been reported that the strongest correlation in estimating age is the condylion–gnathion distance.^29^

In the present study, a comprehensive analysis of the mandible was performed through an advanced shape analysis known as GM analysis. A total of 27 landmarks were placed on each morphology of the mandible in the DPT radiographs. In contrast, another study has used only one parameter, the mandibular angle, on 3D-CT images.^30^ Consequently, age estimation was challenging to confirm because of a tendency to produce estimation errors, and several additional parameters were added to decrease the error in age estimation.^30^

The mandible was used in this investigation because it is the most robust and long-lasting facial bone, which maintains its shape best and frequently withstands post-mortem trauma. It has a unique morphology and changes over the course of life. The mandible has several landmarks that correlate with an individual's age. A preliminary comparative study has indicated that the mandible can feasibly be used to estimate age, sex, and race, with an accuracy as high as 88%.^31^ The mandible is among the types of bone that can be used in identification in the forensic field, in addition to the pelvis, skull, and teeth.

Identification of age, sex, race, and ancestry is essential in forensic investigation.^32^ Many methods have been devised to assist forensic teams during procedures, including use of DNA methods, fingerprints, bite marks, and radiographs. These methods can be used to investigate cases and are complementary. A radiograph must be taken as part of the post-mortem examination in forensic practice to identify foreign bodies and fractures. Dental radiographs such as bitewings, periapical radiographs, and panoramic tomograms may be used. A dental panoramic tomogram is a type of radiograph that provides a panoramic view of the maxilla, mandible, and all teeth, whether erupted or unerupted. It provides a 2D image view and a source of biological data applicable to research purposes. The gonial angle, assessed from DPT, has been found to be almost identical to that measured on the dried mandible.^33^ Moreover, all teeth and developing teeth germs can be distinguished in DPT radiographs. Hence, dental maturity can be assessed, and age can be estimated. However, in certain circumstances, DPT radiographs have several limitations, such as a lack of reliability for incomplete or fractured mandibles.

This research study used 2D image radiographs and DPT, which provides an extensive view of the dentition of an individual. However, in certain circumstances, DPT has several limitations that might have affected this study's outcomes. Image detail was diminished with respect to that of intra-oral images, owing to the superimposition of radio-opaque objects and varied magnification. This problem arose during identification and placement of landmarks in the DPT radiographs. Certain landmarks, for example, landmark number 26, which is between the mandibular central incisors, were difficult to identify because of superimposition with supine vertebrae. Another example is landmarks 12 and 13; we experienced difficulties in locating these landmarks consistently and precisely, because the structures of the mental foramen undergo resorption during the aging period. In addition, some landmarks of the mandible bone may not correlate in determining the age of individuals.

Applying the GM analysis method with measured parameters and morphological features can aid in the determination of age, sex, and race from human mandibles.^34^ In general, GM is a technique for describing a shape according to landmark coordinates. The landmarks should be placed precisely in meaningful locations. The variability of the mandible's shape and size was analyzed through MorphoJ software. These variabilities revealed the pattern shape of each group. MorphoJ also enabled quantitative analysis and interpretation of the collected data, such as significant differences in size and shape between groups. IBM SPSS software 26 was used to determine the significance of distances between each landmark.

Previous traditional morphometric methods have involved measuring linear distances, such as length, width and height, areas, angles, and detailed ratios and counts. Subsequently, multivariate statistical tools were developed to differentiate shape variation among groups. This method is straightforward to use but has some disadvantages. The main disadvantage of linear distance measurement is that it represents the size, not the shape.^35^ Hence, a graphical representation of the shape cannot be reconstructed. Second, linear measurements of two different shapes may yield the same results, because the data do not include the exact landmarks measured. To overcome these problems, GMs was developed. GM on teeth has been studied to estimate age. Tooth size, shape, and morphology have been analyzed, and significant differences have been observed among various shapes and sizes of teeth.^36^

In the forensic, legal, anthropologic, and civil fields, age estimation is crucial.^21^ Age estimation in bones has been performed with a variety of methods. Accurate age assessment is critical in forensics for unidentified remains, bodies recovered from disasters, refugees, and asylum seekers without identifying documents, as well as for legal considerations. Physical bone examination or radiological techniques can be used to estimate age. Various methods have been used, including X-rays, CT scans, ultrasounds, and magnetic resonance imaging.^37^, ^38^, ^39^ Morphological methods, based on radiological examination of dental and skeletal development, have typically been used for forensic age estimation.^6^

Estimation of age in adults may be complicated, because most teeth have already erupted, and the involvement of biological and environmental factors complicates the analysis. The population-specific reference standard used for age estimation in each population is important for accuracy.^20^ Facial skeletons continue to grow throughout life, and the different areas of the face grow at varying rates. Clinical observations suggest that, in some individuals, the shape of the mandible may change with age. Any changes in the size and shape of the mandible (lower jaw) have tremendous importance in cosmetic surgery.^40^

We believe that analysis of more samples may improve the outcomes of this study. The variability of each sampling distribution decreases as the sample size becomes increasingly leptokurtic. Furthermore, 3D radiography provides higher-quality images that may aid in identifying and locating landmarks on the mandible bone in CBCT radiographs. These images have good magnification and diminished geometric distortion, and provide 3D views. Furthermore, analysis of the mandible alone is reliable in determining age and should be used together with another indicator. Finally, in this study, the mandible samples used showed the complete normal morphology. However, this method would have limitations in the analysis of incomplete or missing mandibles. Hence, the odontometric method can be used, because teeth are the most resilient and durable structures in the body, and can withstand high temperatures and bacterial decomposition.

## Conclusion

This study assembled a database from the USIM dental clinic. Numerous landmarks from DPT were evaluated. A simple, non-destructive, and non-invasive tool is described that incorporates a state-of-the-art method consistent with the most current, mature theory of shape analysis. Consequently, it indicates many variations in mandible shape and size, and enables classification according to similar patterns, thus making age estimation possible. We hope that the results of this research study will aid in forensic odontology investigations when patient identification is lacking.

## Source of funding

The research was funded by the Faculty of Dentistry, 10.13039/501100010187Universiti Sains Islam Malaysia (USIM) under an undergraduate research final year project. Encik Malvin and Encik Hafiz helped in collecting data. Grant Research Code: PPP/URG-001.

## Conflict of interest

The authors have no conflict of interest to declare.

## Ethical approval

Ethical approval was obtained from the Ethics Committee, USIM (number USIM/JKEP/2020-90).

## Consent

Not applicable.

## Authors' contributions

NAFZ, NASMS, and AA conceived and designed the study, conducted research, provided research materials, and collected and organized data. NAFZ, NASMS, AA, NMI, CKW, AK and BFWRP analyzed and interpreted data. NAFZ and NASMS wrote the initial and final drafts of the article and provided logistic support. All authors have critically reviewed and approved the final draft and are responsible for the content and similarity index of the manuscript.

## Acknowledgment

We acknowledge Encik Malvin and Encik Hafiz (radiographer) for helping with data collection.

## Funding: research involving human participants and/or animals

Retrospective DPT data were obtained from the Dental Clinic Faculty of Dentistry, USIM.

## Data availability statement

This was a retrospective study conducted at the Faculty of Dentistry, Universiti Sains Islam Malaysia (USIM), which does not require any written informed consent. The patient ID was replaced with a unique ID. No personal details were exposed during the execution of this research. This research was approved by the Research and Ethics committee of USIM. The ethical approval code for this study was USIM/JKEP/2020-90 from USIM.
